# Identification and Management of Differentiation Syndrome in Emergency Settings: A Narrative Review

**DOI:** 10.3390/cancers18111798

**Published:** 2026-06-01

**Authors:** Gregory A. Chang, Tareg Bey, John Stroh, Aiham Qdaisat, Sai-Ching J. Yeung

**Affiliations:** 1 Department of Emergency Medicine, The University of Texas MD Anderson Cancer Center, 1515 Holcombe Blvd., Houston, TX 77030, USA; changg@mskcc.org (G.A.C.); tabey@mdanderson.org (T.B.); jjstroh@mdanderson.org (J.S.); aqdaisat@mdanderson.org (A.Q.); 2 Emergency Care Service, Memorial Sloan Kettering Cancer Center, 1275 York Avenue, New York, NY 10065, USA

**Keywords:** differentiation syndrome, emergency, leukemia, glucocorticoid, complications

## Abstract

**Background:** Differentiation therapy is a cornerstone in treating hematologic malignancies, particularly acute myeloid leukemia (AML). Differentiation agents target molecular defects blocking myeloid differentiation. However, rapid differentiation can precipitate a life-threatening complication, differentiation syndrome (DS). DS manifests with fever, pulmonary infiltrates, pleural or pericardial effusions, hypotension, and organ dysfunction, often mimicking sepsis or infection. Early recognition in the emergency department (ED) is critical to mitigate morbidity and mortality. This review aims to provide emergency clinicians with practical strategies for the timely identification and management of DS in patients undergoing differentiation therapy. **Discussion**: Suspicion for DS should be heightened in patients with acute promyelocytic leukemia (M3 AML) who recently started induction chemotherapy, including all-trans retinoic acid or arsenic trioxide, and in those with non-M3 AML receiving differentiation agents (i.e., isocitrate dehydrogenase inhibitors, menin inhibitors, FMS-like tyrosine kinase 3 inhibitors). Imaging can identify pulmonary infiltrates, effusions, and other cardiopulmonary manifestations. Laboratory workups should include complete blood counts with differentials, serum chemistries, cardiac biomarkers, and sepsis panels to exclude infection. Electrocardiography is advised for patients on QT-prolonging agents. Management emphasizes prompt initiation of high-dose corticosteroids and supportive measures such as blood pressure support, intravascular volume optimization, and oxygen therapy or ventilatory support. Multidisciplinary coordination with oncology, hematology, and critical care teams is important to tailor plans and monitor complications. **Conclusions**: DS represents a diagnostic challenge in the ED due to its nonspecific presentation and mimicry of infection. A high index of suspicion, combined with targeted imaging, laboratory evaluation, and early corticosteroid therapy, can improve outcomes.

## Simple Summary

Differentiation therapy works by turning cancer cells into mature cells that no longer grow, yet this rapid process can provoke a harmful reaction called differentiation syndrome (DS). When this condition happens, usually after starting certain drugs and mainly in patients with hematologic malignancies, these patients may come to the emergency room with symptoms that look like a serious infection, such as fever, trouble breathing, and low blood pressure. In this review article, we highlight that recognizing DS early in emergency departments through physical examination, laboratory results, imaging studies, and starting corticosteroid treatment early can help patients recover and lower the risk of serious complications. We also outline useful current steps that help emergency department physicians in the quick identification and proper management of DS.

## 1. Introduction

Differentiation therapy for cancer is the use of drugs to induce malignant cells to mature into non-proliferative, terminally differentiated cells, particularly in hematologic malignancies such as acute myeloid leukemia (AML). This approach targets defects in biological pathways that cause a pathologic block in cellular differentiation. The rapid differentiation of malignant cells can lead to the release of toxic substances into the circulation, causing adverse systemic responses that can be life-threatening.

Acute promyelocytic leukemia (APL), the M3 subtype in the French-American-British classification system, is a subtype of AML cytogenetically defined (consistent with the current World Health Organization [WHO] and the International Consensus Classification [ICC] classifications) by the t(15;17)(q24;q21) chromosomal translocation, which forms the PML-RARA fusion gene. In 1992, Frankel et al. first described a distinctive clinical syndrome in patients with APL that developed shortly after they had started treatment with retinoic acid [1]. Initially named “retinoic acid syndrome” after the drug causing it, the syndrome is now called “differentiation syndrome” (DS) because its etiology is related to the differentiation of immature myelocytic progenitor cells. The patients Frankel et al. described presented with fever, dyspnea, and hypotension without a clear source of infection. As clinical experience with DS has accumulated, it has become clear that DS’s clinical manifestations typically include hemodynamic instability, weight gain of more than 5 kg secondary to edema, acute renal failure, pulmonary infiltrates, and pleural and pericardial effusions [2]. In the past decade, differentiation therapy using new agents in other subtypes of AML has rapidly advanced. The new classes of anti-leukemia agents can induce differentiation of leukemic cells, leading to DS in clinical scenarios distinct from the familiar setting of induction chemotherapy for APL.

In emergency medicine, early recognition of potentially fatal diagnoses is critical to the successful management of patients. For cancer patients, this is especially true, as many conditions have a high likelihood of rapid decompensation if not addressed appropriately. DS is a prime example of a condition that mimics multiple disease processes, including heart failure, pneumonia, and sepsis, but if recognized immediately, mortality can be reduced from 30% to less than 1% with the early initiation of corticosteroids [3].

Staying abreast of new treatment regimens and recognizing potentially fatal reactions to those treatments is critical for providers caring for patients with AML. In this narrative review, we discuss the pathophysiology and pathogenic mechanism of DS, provide an updated list of clinical scenarios in which DS occurs (i.e., the type of hematologic malignancies, implicated therapies, and timing of DS occurrence relative to initiation of the respective therapies), highlight updates on the diagnostic criteria for DS, and outline the diagnostic workup and clinical management of the complication.

## 2. Materials and Methods

Literature searches were performed in PubMed using the terms “differentiation syndrome” or “retinoic acid syndrome.” The searches were limited to literature in English. The search results were reviewed for relevance and duplication. The articles’ bibliographies and reference sections were reviewed for the inclusion of additional articles as appropriate. Additionally, we reviewed the safety warnings on the package inserts of the involved agents marketed in the U.S. and the listed adverse events in phase I to III trials of each of the drugs discussed.

## 3. Discussion

### 3.1. An Overview of DS

DS is a potentially life-threatening complication of AML treatment and occurs in patients treated with differentiating agents. Classically, DS occurs in patients with APL treated with all-trans retinoic acid (ATRA) and arsenic trioxide (As_2_O_3_) in induction chemotherapy, and complications from DS may contribute to early mortality (i.e., death within 30 days after DS diagnosis) in these patients [4]. The timing of DS presentation is disease-dependent, with DS presenting earlier in the course of induction chemotherapy in patients with APL than during targeted therapy (e.g., therapy with isocitrate dehydrogenase (IDH) inhibitors or menin inhibitors) for patients with other subtypes of AML [5]. DS is characterized by a combination of symptoms, including unexplained fever, dyspnea, pulmonary infiltrates, pleural or pericardial effusions, hypotension, weight gain, peripheral edema, and acute renal dysfunction (see diagnostic criteria below). 

### 3.2. Pathophysiologic Mechanisms

The pathogenesis of DS involves a systemic inflammatory response triggered by the rapid differentiation and activation of leukemic blasts to differentiated mature myelocytes. This leads to uncontrolled cytokine and chemokine release and capillary leakage. On post-mortem examination of patients with DS, Frankel et al. found leukemic infiltrates of maturing myeloid cells in multiple organs, including the kidney, pericardium, liver, and lung [1]. Other studies have also supported the idea that DS is caused by the infiltration of maturing leukemic cells into the lungs and other organs following treatments that induce differentiation [6,7,8,9]. Dense infiltration by differentiating myeloid cells into affected organs can be confirmed by histopathology and immunohistochemistry.

The differentiation of myeloid cells upregulates the expression of adhesion molecules (such as leukocyte function-associated antigen 1 [LFA-1] and intercellular adhesion molecule 2 [ICAM-2]), promoting cellular aggregation and tissue infiltration, especially in the lungs [6,9,10,11,12]. DS is increasingly recognized as a cytokine-driven inflammatory process triggered by rapid leukemic cell maturation following differentiating therapy. Differentiating agents, including ATRA and arsenic trioxide, are associated with increased production of inflammatory cytokines and chemokines that contribute to endothelial injury, vascular permeability, systemic inflammation, and myeloid tissue infiltration. Chemokine and cytokine production (e.g., C-C motif chemokine ligand [CCL]2, CCL24, interleukin [IL]-1 beta [IL-1β], IL-6, IL-8, and tumor necrosis factor alpha [TNFα]) are markedly increased [7,13], driven by activation of nuclear factor-kappa B (NF-κB) signaling and the aberrant expression of transglutaminase 2 (TG2) in differentiating cells [7,14,15]. Local and systemic cytokine release causes endothelial dysfunction (e.g., the direct effects of inflammatory mediators on endothelial tight junctions) and increased capillary permeability, leading to the leakage of protein-rich fluid from the intravascular space into the interstitial compartment and clinical manifestation as edema, effusions, and pulmonary infiltrates. Inflammatory cytokines increase intracellular calcium and the activation of myosin light chain kinase (MLCK) and Ras homolog family member A (RhoA), leading to actin cytoskeletal contraction, phosphorylation, and the consequent endocytosis of tight-junction proteins (e.g., occludin and claudins), and the opening of paracellular gaps [16,17,18,19]. In DS, the inflammatory milieu leads to increased production of reactive oxygen species by the infiltrating myeloid cells. Oxidative stress generated by reactive oxygen species further modifies tight-junction proteins through thiol oxidation, phosphorylation, and ubiquitination, destabilizing the junctional complexes and increasing permeability [18]. Vascular endothelial growth factor [VEGF], which may be upregulated by the cytokines, may also promote occludin phosphorylation and ubiquitination, leading to endothelial cell tight-junction dysfunction [20]. The dysregulated activation of the inflammatory cascade manifests clinically as fever, hypotension, dyspnea or respiratory distress, pulmonary infiltrates, pleural or pericardial effusions, and renal failure [21,22]. Given that the white blood cell (WBC) count may reflect the burden of leukemic disease, leukocytosis may predict a high level of tissue injury by increasing the burden of infiltrating cells, and it is a strong risk factor for both the risk of DS occurrence and of DS severity [23,24,25]. Additionally, the inflammatory mechanisms also provide the biologic rationale for glucocorticoid-based therapy and anti-cytokine therapeutic strategies aimed at suppressing cytokine signaling to mitigate or limit endothelial injury, capillary leak, and organ dysfunction.

### 3.3. Leukemia Therapies That Can Cause DS and Clinical Scenarios

Information on the incidence rates and timing of DS in patients with AML is provided in Table 1.

#### 3.3.1. Induction Chemotherapy for APL Using ATRA or As_2_O_3_

The introduction of vitamin A-related compounds (retinoids) and As_2_O_3_ has revolutionized the treatment of APL by turning pathological leukemic cells into terminally differentiated myeloid cells via completion of the maturation process. Following the widely accepted use of ATRA and As_2_O_3_, the remission rate of APL has reached 90–95% [27,28,29]. The rationale for the use of retinoids and As_2_O_3_ is based on the t(15;17)(q24;q21) chromosomal translocation in APL. This chromosomal abnormality in myelocyte precursors leads to the production of a fusion protein, promyelocytic leukemia (PML)-retinoic acid receptor alpha (RARA), which silences the genes that promote differentiation. ATRA counteracts the action of PML-RARA by binding to the RARA moiety, inducing a conformational change that releases corepressor complexes and recruits coactivators, thereby restoring the transcription of genes required for myeloid differentiation [30,31,32]. On the other hand, As_2_O_3_ directly binds to the PML moiety, inducing its SUMOylation, polyubiquitination, and subsequent proteasome-mediated degradation [33,34,35]. As_2_O_3_ induces partial differentiation of APL cells at low concentrations (0.1–0.5 μM) but leads to predominantly apoptosis at high concentrations (0.5–2 μM). Although As_2_O_3_ may disrupt PML nuclear bodies to mitigate the oncogenic functions of PML-RARA, it primarily induces post-transcriptional modifications leading to the degradation of PML-RARA [33,36].

The incidence rate of DS in patients with APL treated with ATRA without As_2_O_3_ is 15–25% [37,38,39]. A recent clinical trial demonstrated that the incidence of DS during induction with ATRA and As_2_O_3_ is 24.5% in standard-risk APL and 30.4% in high-risk APL [40]. Overall, the real-world incidence of DS in APL treated with ATRA and As_2_O_3_ ranges from 19.9% to over 40%, depending on the APL risk category, the DS diagnostic criteria, and the use of prophylactic corticosteroids [41,42]. In a review of DS incidence in 39 clinical trials, Gasparovic et al. noted that the weighted mean incidence of DS ranged from 15.4% to 20.6% for any combination of ATRA or As_2_O_3_ with cytotoxic therapy [38]. However, after accounting for potential biases in 7 trials, the range of the weighted mean incidence rose to between 17.4% and 29.3% [38].

In a study of 183 cases [39], DS occurred in a bimodal distribution after ATRA initiation. The first peak, accounting for almost half of the DS cases, was in the first week of ATRA treatment. Only 5% of the DS cases occurred during the second week, and the second peak of DS occurrence was in the third week. The latest DS occurrence in the study was 46 days after ATRA initiation. Therefore, it appears that most cases of DS in patients with APL occur while the patients are still hospitalized. However, delayed occurrence of DS in a significant portion of APL patients undergoing induction chemotherapy can occur after discharge, and these patients will probably present to EDs. When stratified by severity, the median number of days to DS presentation after starting induction therapy is 6 days for severe DS and 15 days for moderate DS [39]. It is noteworthy that the late occurrence of severe DS is associated with a higher frequency of hypotension than the early occurrence of severe DS [39]. There is no consensus on the prophylactic use of glucocorticoids to prevent DS; DS can occur despite prophylaxis and can recur upon restarting ATRA after stopping it due to an episode of DS [43]. DS is uncommon later in the treatment course (particularly beyond 6 weeks after starting induction chemotherapy), including during the consolidation and maintenance periods [37,44].

#### 3.3.2. Bexarotene

Like retinoic acid receptors, retinoid X receptors also regulate cellular differentiation. Bexarotene, a synthetic retinoid classified as a rexinoid, selectively binds and activates retinoid X receptors (RXRα, RXRβ, and RXRγ). This activation modulates gene expressions involved in cellular differentiation, proliferation, and apoptosis. Bexarotene is approved for the treatment of cutaneous T-cell lymphoma. It is rarely used for AML, but due to its classification as a retinoid-receptor agonist, it has been investigated for use in relapsing and refractory AML [45,46,47,48]. In a review of trial treatments using bexarotene and tamibarotene (another retinoid X receptor agonist), the mean incidence of DS was 5.9% [38]. In a case report by Dinardo et al., bexarotene caused DS in a non-M3 AML patient 36 days after treatment initiation [48]. Although there were a few early-phase clinical trials of bexarotene that showed some utility in this patient group, toxicities and the heterogeneity of AML limit its value because of the lack of a clear target. To date, there have been no new, large-scale clinical trials exploring bexarotene as therapy for AML and no smaller-scale studies since 2015.

#### 3.3.3. IDH Inhibitors

IDH plays a key role in cellular respiration as a nicotinamide adenine dinucleotide phosphate-dependent enzyme. About 15–20% of AML patients harbor mutations in IDH1 or IDH2, resulting in the increased production of 2-hydroxyglutarate (2HG) via a neomorphic activity that uses α-ketoglutarate as the substrate. 2HG inhibits the ten-eleven translocation (TET) family of methylcytosine dioxygenases (primarily TET2 in hematopoietic cells); these methylcytosine dioxygenases catalyze the oxidation of 5-methylcytosine to 5-hydroxymethylcytosine, initiating active DNA demethylation. Therefore, the oncometabolite 2HG leads to DNA hypermethylation [49,50], and thus impairs cellular differentiation, induces abnormal epigenetic regulation of myeloid cells, and contributes to leukemogenesis [51,52,53,54].

Currently, ivosidenib, olutasidenib, and enasidenib are approved by the US FDA for the treatment of IDH-mutated AML. Ivosidenib is approved for adults with newly diagnosed IDH1-mutated AML who are ≥75 years old or have comorbidities precluding intensive induction chemotherapy, either as monotherapy or in combination with azacitidine, and for relapsed or refractory (R/R) IDH1-mutated AML [52,53,55]. Olutasidenib and enasidenib are approved for the treatment of R/R IDH1-mutated AML and IDH2-mutated AML, respectively [56,57,58,59].

IDH inhibitors suppress 2HG production and thus enable cell differentiation to proceed [60]. Like other therapies that allow proper cell differentiation to resume in neoplastic cells, IDH inhibitors have been reported to cause DS. The overall incidence rate of DS is approximately 6–25% for IDH inhibitors, with rates of 3.9–25% for ivosidenib-treated patients, 16% for olutasidenib-treated patients, and 6–19% for enasidenib-treated patients [2,53,57,61,62,63,64,65].

The incidence rate of DS induced by IDH inhibitors in patients with non-M3 AML appears to be lower than that of DS induced by ATRA and As_2_O_3_ in patients with APL. A systematic review found that the incidence rates were 5.9% and 10.4%, respectively [38]. In contrast to the aforementioned early occurrence (short median time after initiation of therapy) of DS in patients with APL after induction chemotherapy with regimens containing ATRA and As_2_O_3_, the median time to DS presentation in patients with non-M3 AML was 20–29 days after starting ivosidenib, 17.5 days after starting olutasidenib, and 19–30 days after starting enasidenib. However, cases occurred as late as 78 days, 561 days, and 150 days after treatment initiation, respectively [2,57,62]. Therefore, most cases of DS in patients with non-M3 AML treated with IDH inhibitors occur on an outpatient basis, and patients will most likely present to the ED. Missed diagnoses of DS after the administration of enasidenib have led to fatalities [62]. Therefore, the prompt diagnosis and proper management of DS in patients treated with IDH inhibitors is highly important.

#### 3.3.4. Menin Inhibitors

AML subtypes with lysine methyltransferase 2A (KMT2A) gene rearrangements, nucleoporin 98 (NUP98) fusion, and nucleophosmin 1 (NPM1) mutations have leukemogenic gene expression requiring protein–protein interaction between menin and KMT2A, in which menin serves as a chromatin adapter and facilitates the recruitment of KMT2A fusion proteins to sustain the transcription of genes driving the transformation and blocking the differentiation of immature myeloid cells (e.g., HOXA9, MEIS1, and PBX3). KMT2A mutations lead to partial tandem duplication or gene rearrangement, which, in turn, lead to overexpression of the HOXA9 and MEIS1 genes [66,67]. Gene rearrangement of KMT2A leads to fusion proteins, including AF9, which is the fusion of the KMT2A and MLLT3 genes. NPM1 is a regulator of genome stability and a tumor suppressor, and NPM1 mutations are also directly involved in the overexpression of HOXA9 and MEIS1 via abnormal activation of the menin pathway [68]. The frequencies of KMT2A rearrangement, NUP98 fusion, and NPM1 mutations are approximately 4–10%, 1–2%, and 30%, respectively, in adult patients with AML. Blocking the menin-KMT2A axis inhibits AML with these gene abnormalities by disrupting a critical transcriptional program that maintains leukemogenicity, leading to terminal differentiation of leukemic cells.

Revumenib is a menin inhibitor approved by the US Food and Drug Administration for the treatment of adults with R/R AML harboring a KMT2A rearrangement [69,70]. Menin inhibitors block the formation of the menin-KMT2A complex, thus downregulating the transcription of downstream targets and leading to differentiation of abnormal leukemic cells [71]. As expected, the increase in differentiation leads to DS. In the AUGMENT-101 phase 2 study of revumenib, the incidence rate of all-grade DS was 28%, and the incidence of grade 3 or higher DS was 16% [72]. Other menin inhibitors are in development and undergoing clinical trials to evaluate their safety profiles. Phase 1 studies of two of these inhibitors, ziftomenib and JNJ-75276617, had all-grade DS incidence rates of 21.7% [73] and 14% [74], respectively. A current list of menin inhibitors that are under investigation or are FDA-approved has been compiled in a recent publication [75].

#### 3.3.5. FMS-like Tyrosine Kinase 3 Inhibitors

FMS-like tyrosine kinase 3 (FLT3) is a tyrosine kinase receptor in hematopoietic cells. Internal tandem duplications in the juxtamembrane domain or point mutations of FLT3 that cause constitutive activation of tyrosine kinase activity. Mutations in FLT3 trigger the activation of several downstream signaling pathways, including the signal transducer and activator of transcription 5, mitogen-activated protein kinase/extracellular signal-regulated kinase, and phosphoinositide 3-kinase/protein kinase B pathways. This activation leads to the autonomous proliferation and survival of leukemia cells, hinders their ability to differentiate properly, and results in a high leukemic burden. FLT3 mutations are found in 20–30% of all patients with AML and in up to 40% of younger patients [76]. FLT3 inhibition can lead to terminal myeloid differentiation of FLT3-mutated AML [77,78]. Because of the high risk of relapse and the low cure rates among patients with FLT3-mutated AML, the push to develop therapies against FLT3 mutations has led to several generations of inhibitors. First-generation multi-kinase inhibitors include sorafenib, lestaurtinib, and midostaurin, and later-generation inhibitors include quizartinib, crenolanib, and gilteritinib [79]. Overall, the incidence rates of DS in patients treated with FLT3 inhibitors are about 1–5% [78,80,81]. In contrast with DS in patients with APL, FLT3 inhibitors induce apoptosis in a large proportion of FLT3/ITD leukemic cells with gradual differentiation of the remaining myeloblasts, and the surge in the absolute neutrophil count after leukemic-cell differentiation is modest [77]. Surprisingly, no cases of DS have been reported with midostaurin to date. DS occurs in up to 3% of patients on gilteritinib [78,81], and it occurs from 2 to 75 days after starting treatment with the drug [81]. DS occurs in up to 5% of patients on quizartinib [82], and it occurs from within days to 6–8 weeks after starting treatment [77].

#### 3.3.6. Azacitidine

Azacitidine is a cytidine analog that is incorporated into DNA and RNA and causes inhibition of DNA and RNA methyltransferases, leading to hypomethylation of DNA and RNA, altered gene expression, and direct cytotoxicity to abnormal hematopoietic cells. Azacitidine reduces DNA methylation, reactivates genes involved in tumor suppression and cell differentiation, and can cause DS in patients with AML. A case report described a patient with AML who developed a clinical syndrome resembling DS during initial treatment with azacitidine and who improved rapidly after corticosteroid therapy [83]. In a clinical trial, DS occurred in both patients receiving ivosidenib plus azacitidine and in those receiving a placebo plus azacitidine [52]. The incidence of DS in the azacitidine group was lower than in the ivosidenib plus azacitidine group (8% vs. 15%). These data indicate that azacitidine, even in the absence of other differentiating agents, can precipitate DS. The mechanism is not fully understood, but clinicians should be aware of this potential adverse event when initiating azacitidine, especially in patients with AML. Prompt recognition of DS in these patients is important, and management with glucocorticoids and supportive care is recommended.

### 3.4. Diagnostic Criteria for DS

Historically, DS was recognized in a small set of APL patients treated with ATRA whose clinical presentation and imaging findings primarily indicated a systemic inflammatory response syndrome (SIRS) and a “capillary leak” syndrome [1]. In the National Cancer Institute’s Dictionary of Cancer Terms, the signs and symptoms of DS include “fever; cough; trouble breathing; weight gain; swelling of the arms, legs, and neck; build-up of excess fluid around the heart and lungs; low blood pressure; and kidney failure” [84]. In a prospective clinical trial with the incidence of DS as the primary study end point, Montesinos et al. [39] made the diagnosis of DS according to the presence of “dyspnea, unexplained fever, weight gain greater than 5 kg, unexplained hypotension, acute renal failure, and, particularly, a chest radiograph demonstrating pulmonary infiltrates or pleuropericardial effusion” (See Table 2). The authors also stated, “Patients with 4 or more of the above signs or symptoms were classified as having severe DS, while those with 2 or 3 signs or symptoms were considered to have moderate DS. No single sign or symptom was considered sufficient to make a diagnosis of the syndrome” [39]. Grading can also be done based on symptomology using Common Terminology Criteria for Adverse Events version 5.0 (CTCAE v5). In recent studies, the common signs and symptoms of DS in descending order are dyspnea and pulmonary infiltrates (50–97%), fever (79%), pleural effusions (30–50%), pericardial effusions (30–52%), and renal failure (10–40%) [85,86]. Less common findings include weight gain (17%), cardiac failure (15–20%), and hypotension (10–17%) [87].

Laboratory abnormalities in patients with DS often include leukocytosis (>10 × 103/μL; prevalence, 50%; values > 30 × 103/μL, associated with early mortality); elevated creatinine (prevalence, 11–66%); hyperbilirubinemia (21%); and elevated troponin or brain natriuretic peptide (BNP) levels [1,23,37,87,88,89,90,91]. An elevation in creatinine indicative of acute kidney injury (a rise in serum creatinine of ≥0.3 mg/dL within 48 h or ≥50% above baseline within 7 days) [92] is a criterion for DS.

There are no pathognomonic radiographic findings for DS. However, the most common findings are cardiopulmonary (in 38% of patients with moderate DS and 80% of patients with severe DS) and include cardiomegaly, widening of the vascular pedicle, signs of pulmonary vascular congestion, peribronchial cuffing, ground-glass opacities, and pleural effusions [91,93,94]. Small, irregular peripheral nodules in the lungs and pleural effusions are the most consistent computed tomography findings in DS [95]. Ultrasound of the chest has a high sensitivity and specificity for alveolar consolidation, interstitial syndrome, pulmonary edema, pleural effusion, and pericardial effusion [96], which may be present in DS. Currently, there is a paucity of literature about the use of diagnostic ultrasound in the evaluation of DS, although a small prospective study demonstrated the feasibility of using point-of-care chest ultrasounds to detect pulmonary abnormalities in DS and was able to identify the “comet-tail sign” 12 h prior to the onset of symptoms in one out of three patients who developed DS [97].

Acute febrile neutrophilic dermatosis (AFND), also known as Sweet syndrome, is a rare inflammatory disorder characterized by the acute onset of painful, erythematous skin lesions (papules, plaques, or nodules), commonly on the upper limbs, trunk, neck, and face [98]. In the AML population, AFND can present either as a paraneoplastic phenomenon or as a drug-induced dermatosis, often in response to agents inducing myeloid cell differentiation such as ATRA, FLT3 inhibitors, or IDH inhibitors. AFND may occur concurrently with DS or in isolation, and cases have been reported to mimic cellulitis, necrotizing fasciitis, and disseminated fungal infections [99,100]. Ghiaur et al. pointed out that patients with APL mostly exhibited dermatological side effects during induction chemotherapy with both arsenic trioxide and ATRA, but with lower rates being reported during consolidation or maintenance cycles [101].

Because AFND and DS can occur concurrently and share the manifestation of SIRS in patients with AML, the presence of one of these conditions should prompt a thorough evaluation to look for the other.

Even in patients who have recently been treated with a differentiating agent, a presumptive diagnosis of DS made on the basis of just a single sign or symptom is controversial. Making the diagnosis based on a single symptom risks misattribution and inappropriate management. Therefore, careful clinical judgment and comprehensive assessment for multiple concurrent features are essential for accurate diagnosis and optimal treatment. After excluding other possible causes, DS can be diagnosed. However, identifying DS continues to be a challenge, as rare presentations such as atypical cardiac symptoms (pericarditis or myocarditis) [102,103,104], pancreatitis, hyperbilirubinemia, myalgia, painless genital ulcers [105], painful oral ulcers [106], and ocular manifestations (subretinal fluid, retinal detachment, macular edema, or exudative hemorrhage retinopathy) [107,108,109,110] are also possible [103,107,108,110,111,112,113].

### 3.5. Diagnostic Workup for, Management of, and Prognostic Factors for DS

**Figure 1:**
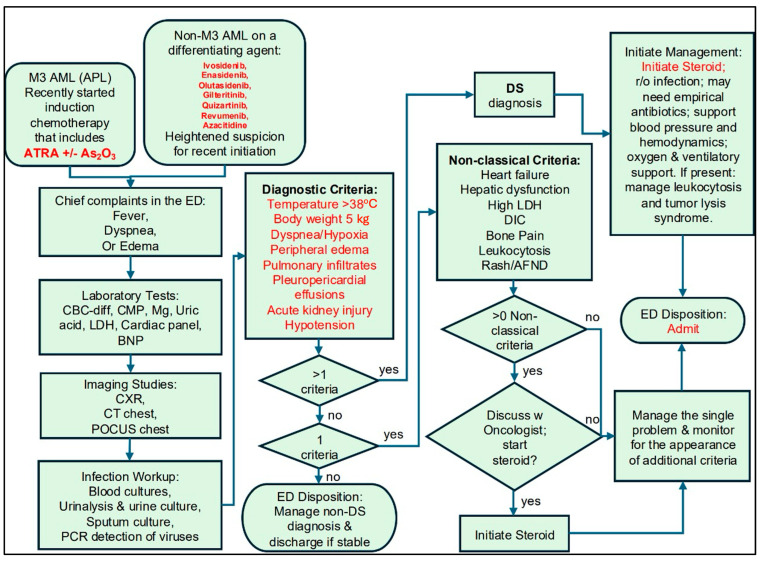
An algorithm for the diagnosis and management of differentiation syndrome in the emergency department. AFND, acute febrile neutrophilic dermatosis; AML, acute myeloid leukemia; APL, acute promyelocytic leukemia; ATRA, all-trans retinoic acid; BNP, brain natriuretic peptide; CBC-diff, complete blood count with differential; CMP, comprehensive metabolic panel; CT, computed tomography; CXR, chest x-ray; DIC, disseminated intravascular coagulation; DS, differentiation syndrome; ED, emergency department; LDH, lactate dehydrogenase; Mg, magnesium; PCR, polymerase chain reaction; POCUS, point-of-care ultrasound; r/o, rule out.

Fever, dyspnea, and edema are commonly the complaints that prompt patients with possible DS who are not hospitalized to present to the ED. The first step is to determine whether the patient in the ED is at risk for DS (Figure 1). The patients at risk fall into two main groups. The first group is patients with APL who have recently been discharged after hospitalization for induction chemotherapy and are currently on ATRA or As_2_O_3_. DS occurs within 12 days after starting ATRA or As_2_O_3_ in about half of the cases but can occur as late as 46 days after starting induction chemotherapy [39]. Although there is no consensus regarding glucocorticoid prophylaxis for DS during induction chemotherapy for APL, some patients with APL, particularly those with high leukocyte counts [93], may be taking 0.5 mg/kg of prednisone for the duration of induction, 1 mg/kg of prednisolone on days 1–10, 20–50 mg/day of methylprednisolone for 5–10 days, or 2.5/mg/m2 of dexamethasone every 12 h on days 1–15 [22,39,114,115,116]. The second group is patients with non-M3 AML who are receiving treatment with the following classes of drugs that can induce differentiation: (1) IDH inhibitors (i.e., ivosidenib, olutasidenib, enasidenib), (2) menin inhibitors (i.e., revumenib), (3) FLT3 inhibitors (i.e., quizartinib, crenolanib, gilteritinib), (4) retinoids or rexinoids (i.e., ATRA, bexarotene), and (5) hypomethylating agents (i.e., azacitidine). DS typically occurs within 19 days after starting an IDH inhibitor but can occur many months after starting the drug [2,57,62]. The timing of DS in patients with non-M3 AML who are taking the other four classes of drugs is not very clear due to a paucity of data, but it may be similar to that for IDH inhibitors.

For patients with AML who fit the clinical scenario for DS occurrence and have signs and symptoms that suggest DS as a differential diagnosis, the diagnosis of DS primarily relies on clinical, laboratory, and radiological findings (Figure 1 and Table 3). The diagnostic workup for a patient initially suspected to have DS involves a rapid, systematic assessment to exclude alternative etiologies (especially infection and heart failure), ascertain the presence of diagnostic criteria for DS, and evaluate severity. 

### 3.6. Evaluation

#### 3.6.1. Characteristic Signs and Symptoms by History and Physical Examination

Signs and symptoms include fever, dyspnea, hypoxia, weight gain, peripheral edema, hypotension, pleural effusions, and pericardial effusion.

#### 3.6.2. Laboratory Studies

A complete blood count with differential can assess leukocytosis and neutrophilia. A complete metabolic panel (including electrolytes and renal and hepatic function tests) can assess acute kidney injury and tumor lysis. Uric acid levels will also need to be checked. Elevated troponin and pro-BNP levels may suggest cardiac involvement or hypervolemia. Assess coagulopathy and disseminated intravascular coagulation if clinically indicated. Cytokine elevations (such as IL-6, IL-1β, and TNFα) may provide mechanistic and prognostic insight for DS. Experience from the COVID-19 pandemic demonstrated the role of cytokines, particularly IL-6, in the development of cytokine storm and highlighted the potential effectiveness of cytokine-directed therapeutic strategies [117,118,119]. Despite current limitations across EDs in assay availability, standardization, and turnaround time, rapid cytokine testing in select ED settings may support earlier recognition of severe disease and guide targeted therapeutic interventions. Additionally, nonspecific readily available inflammatory markers such as C-reactive protein and ferritin may be helpful to assess disease activity, even though these markers are not specific or diagnostic [120]. An elevated WBC count of more than 5–10 cells × 103/μL, poor performance status, an elevated creatinine level, a decreased albumin level, and the presence of coagulopathy have been suggested as possible prognostic factors for DS [39,121,122].

#### 3.6.3. Diagnostic Imaging

Chest radiographs or computed tomography scans can identify pulmonary infiltrates, nodules, peribronchial cuffing, ground-glass opacities, consolidation, pleural effusions, pericardial effusions, cardiomegaly, and increased vascular pedicle widths [93,94,123]. These imaging studies may exclude alternative causes of respiratory symptoms (e.g., lung abscess, pulmonary hemorrhage, chronic pulmonary obstructive disease). Point-of-care ultrasound may be a valuable tool for evaluating cardiopulmonary pathology in patients with suspected DS in the ED, particularly to detect pulmonary and serosal manifestations such as pleural and pericardial effusions and pulmonary edema [96,97]. However, it cannot be a standalone diagnostic tool for DS because these cardiopulmonary abnormalities must be interpreted in the complete clinical context.

#### 3.6.4. Microbiologic Studies

Standard sepsis workups may be appropriate, as the initial presentations of DS are often categorically SIRS-positive. The diagnostic workup should include blood cultures, urine cultures, and sputum cultures as DS can mimic infection or sepsis [26]. Detection of viral infections (e.g., Epstein–Barr virus, cytomegalovirus) using polymerase chain reaction may be considered if clinically indicated.

#### 3.6.5. Cardiac and Renal Assessment

Echocardiograms, cardiac panels, BNP, or NT-proBNP if there is evidence of weight gain, edema, effusions, hypotension, or renal impairment. An electrocardiogram should be obtained to check the corrected QT duration in patients who are on drugs that prolong the QT interval (e.g., quizartinib, midostaurin, gilteritinib, glasdegib, enasidenib, ivosidenib, As_2_O_3_, voriconazole, posaconazole, fluconazole, macrolides, fluoroquinolones, prochlorperazine, and ondansetron) and have electrolyte abnormalities.

### 3.7. Treatment and Management

Early recognition and prompt treatment are crucial for reducing morbidity and mortality associated with DS. Additionally, although the underlying pathophysiologic cascade may overlap, DS related to novel non-M3 AML therapies often introduces management challenges distinct from those encountered in APL-associated DS [2], necessitating different treatment interruption thresholds, monitoring strategies, and supportive care approaches. In APL-associated DS, management has evolved into a relatively standardized approach that frequently incorporates corticosteroid prophylaxis during induction, selective continuation or temporary interruption of differentiating therapy based on symptom severity, and predefined cytoreductive strategies [2,22,91,124,125]. Conversely, DS related to newer targeted therapies in non-M3 AML generally remains less protocolized and often necessitates individualized decisions regarding treatment interruption, duration of corticosteroid therapy, and escalation of supportive or cytoreductive interventions [2,26,126]. Main treatment options (Table 3) include the following:

#### 3.7.1. Corticosteroids

Initiation: The cornerstone of management for DS is early initiation of high-dose steroids, typically intravenous dexamethasone (10 mg every 12 h) to be started as soon as DS is diagnosed or at the earliest clinical suspicion of incipient DS in patients with APL [124]. If this dose does not improve the patient clinically within 24 h, the dosing frequency may be increased to every 6 h [39,121,124,127,128,129], but alternative diagnostic possibilities must also be considered.

Duration: Continue dexamethasone for at least 3 days or until symptoms resolve.

Tapering: A slow taper of corticosteroids may be considered to prevent rebound symptoms, especially when small-molecule inhibitors are involved due to their longer half-lives.

#### 3.7.2. Cytoreduction for Hyperleukocytosis

Initiate cytoreduction for leukocytosis exceeding 10,000 cells/µL in APL and based on protocol-directed thresholds in other forms of AML.

Typical Agent: Hydroxyurea is a commonly used cytoreductive agent for leukocytosis during treatment. Prior to the initiation of hydroxyurea, ED providers should always consult the primary oncology team to assess the risks and benefits of the therapy. If hydroxyurea is appropriate for the management of DS, then protocols for tumor lysis syndrome may be required. This includes monitoring for dysrhythmias secondary to electrolyte abnormalities, hyperuricemia management, and admission to the intensive care unit. Of note, leukapheresis is not recommended as it is likely to precipitate fatal hemorrhage due to disseminated intravascular coagulation [89,121,124,129].

#### 3.7.3. Differentiating-Agent Modification

Finally, the offending agent should be discontinued immediately in severe cases of DS. Upon resolution of symptoms, the oncologist may restart the agent; however, if DS recurs, the offending agent must be definitively discontinued.

Holding Therapy: Temporarily discontinue the differentiating agent (e.g., ATRA [124], As_2_O_3_ [124], IDH inhibitors, menin inhibitors, and FLT3 inhibitors) in patients with severe DS (e.g., hypoxic respiratory failure, severe renal dysfunction) or symptoms persisting for 48 h despite corticosteroids.

Resuming Therapy: Once symptoms resolve to Grade 2 or less, differentiating agents can be resumed.

Dose Adjustment: ATRA and As_2_O_3_ should be resumed at 50% of the most recently prescribed dose for 7 days, and the dose can be increased to 100% if DS does not recur. Small-molecule inhibitors can be resumed without dose modifications.

#### 3.7.4. Supportive Care

Supportive measures should be started based on the patient’s signs and symptoms.

Respiratory Support: Provide high-flow oxygen therapy and consider invasive or noninvasive mechanical ventilation for severe acute respiratory failure. Intubation and mechanical ventilation may be necessary in cases of severe respiratory failure [91].

Fluid and Electrolyte Management: Carefully administer fluids and vasopressors as needed to manage hypotension and prerenal failure. Fluids should be considered carefully, as many patients with DS may be hypoxic due to fluid overload. On the contrary, intravascular depletion due to third spacing may lead to worsening renal failure. Acute kidney injury is treated with diuretics; however, some patients may require dialysis [130].

Diuretics: These may be used to manage acute renal failure and weight gain.

Infection Prevention: Initiate empiric intravenous antibiotics if infection is a concern.

Avoidance of Invasive Procedures: Generally, avoid invasive procedures for diagnosing pleural and pericardial effusions unless absolutely necessary.

### 3.8. Challenges and Pitfalls in Emergency Department Management of DS

The key prognostic factors that predict the outcome of DS include the DS severity, initial and increasing WBC counts (particularly when the WBC count exceeds 5–10 cells × 103/μL or rises quickly during induction) [23,39,131], renal dysfunction at diagnosis [39], and promptness of corticosteroid administration [2,21,22,26]. Severe DS, marked by symptoms like hypotension, respiratory failure, kidney issues, and multi-organ involvement, leads to higher early mortality rates and lower long-term survival compared with milder DS [21,39].

The quick identification of DS and immediate administration of corticosteroids, along with cytoreductive therapy in cases of hyperleukocytosis, are crucial for improving outcomes [2,21,22,26]. The prophylactic use of corticosteroids in high-risk patients (such as those with elevated WBC counts) can decrease the incidence and severity of DS and enhance survival rates [22,39]. Delays in diagnosis or treatment are linked to increased morbidity and mortality [2,26,39]. The diagnostic criteria based on the work of Montesinos et al. have not been formally tested in patients with non-M3 AML treated with differentiating agents, and experts have suggested devising diagnostic criteria that include the less-common signs and symptoms that were not originally described by Frankel et al. [1] but were used in the work of Montesinos et al. When one criterion is present in a patient that fits the risk profile, the patient should be closely monitored for the occurrence of additional criteria confirming the diagnosis of DS. Since DS cannot be diagnosed with confidence when only one diagnostic criterion is present, an update by an European expert panel recommended starting glucocorticoid therapy at the earliest clinical suspicion of incipient DS in patients with APL [124]. Because the literature is unclear about what to do when one DS diagnostic criterion is present in a patient with non-M3 AML who is on differentiation therapy, the decision to start glucocorticoid therapy in the ED should be made jointly through discussion with the oncology team. It is also crucial to address any other likely or suspected co-existing problems—such as an infection or an exacerbation of congestive heart failure—at the same time, because patients with relapsed or refractory AML often have several different causes contributing to their symptoms.

ED recognition of DS is often hindered by nonspecific clinical manifestations that mimic infectious or cardiopulmonary emergencies [132]. Frequent pitfalls include delayed treatment while awaiting diagnostic confirmation, failure to recognize DS in patients receiving newer oral AML-directed therapies, and underestimation of delayed-onset disease occurring outside the traditional timeframe associated with APL therapy. Early suspicion and timely corticosteroid initiation remain key components of management [133,134]. Moreover, given the substantial overlap between the clinical presentations of DS and sepsis, empiric antibiotics may be administered when infection is a concern, but this should not delay or postpone corticosteroid initiation in suspected DS or when DS is among the differential diagnoses.

## 4. Future Directions

As the use of differentiating therapies broadens beyond classic APL and increasingly shifts toward ambulatory management of non-M3 AML, there remains a critical need to enhance recognition and management of DS in emergency and acute care settings.

### 4.1. Improvement in Precision Diagnostics

Future investigations should explore the utility of biomarkers, cytokine signatures, and imaging modalities (e.g., point-of-care ultrasonography) in differentiating DS from sepsis and other inflammatory or cardiopulmonary conditions, while also improving assessment of disease severity and therapeutic response.

### 4.2. Alternative Therapies and Combination Therapies

For management, recognition of DS as a hyperinflammatory cytokine-mediated state has prompted interest in targeted anti-cytokine interventions for severe or refractory disease. Potential alternative and adjunctive approaches to be combined can aim at reducing circulating inflammatory mediators to mitigate capillary leak and organ dysfunction.

Although the data are sparse in DS, several drugs may be investigated because they target the same cytokine-storm mechanisms seen in cytokine release syndrome (CRS):

- Tocilizumab (an anti-IL-6 receptor antibody),
- Anakinra (an IL-1 receptor antagonist),
- Ruxolitinib (a JAK inhibitor).

Their use in DS may be inferred from the experience with CRS management. Similarly, extracorporeal cytokine-adsorptive therapies also seem reasonable in severe or refractory DS.

Pre-clinical studies suggest that combining ATRA with calcidiol (25-hydroxy vitamin D_3_) reduces NF-κB activation and lowers ATRA-induced cytokine production (IL-1β, TNF-α, MCP-1), dampening the cytokine storm that underlies DS. This approach remains experimental and has not yet been proven in patients [135].

Overall, most interventions beyond corticosteroids have minimal evidence to support their routine use and remain controversial. Further studies are warranted to establish evidence-based approaches for diagnostic and prognostic biomarkers, and the potential role of emerging anti-cytokine and extracorporeal cytokine-directed therapies in severe or refractory DS.

## 5. Conclusions

The early recognition and prevention of DS, a life-threatening emergency, are crucial for the effective management of patients with AML, especially in patients using targeted therapies. Patients should be informed about potential DS symptoms such as unexplained fevers, hemodynamic instability, weight gain from edema, renal failure, and pulmonary infiltrates or effusions, and they should be encouraged to be especially vigilant regarding these symptoms during the first two months of therapy. Although DS is often associated with patients with APL who are undergoing treatments like ATRA and As_2_O_3_, it also occurs in patients with non-M3 AML who are undergoing treatment with IDH inhibitors, menin inhibitors, FLT3 inhibitors, and other differentiating agents (e.g., bexarotene, azacitidine). Prompt initiation of glucocorticoids at the onset of DS can significantly reduce mortality rates to ~1% or less [91]. While the strongest evidence comes from APL-associated DS studies, early glucocorticoid therapy remains a cornerstone of management for DS associated with newer non-M3 AML-directed therapies. At present, the primary strategy continues to be early detection of DS and rapid initiation of dexamethasone, followed by a taper of at least three days after symptoms resolve to help prevent recurrence. To further decrease DS-related morbidity and mortality, emergency physicians, who are at the frontline taking care of patients with DS, need to be equipped with the knowledge of diagnostic and therapeutic strategies.

## Figures and Tables

**Table 1:** Incidence Rates and Timing of Differentiation Syndrome in Patients with AML.

Drug	Mechanism of Action	DS Incidence Rate (%)	Timing in Days, Median (Range) *	Notes
All-trans retinoic acid (ATRA)	Retinoic acid receptor agonist	17.4–29.3	12 (0–46)	Foundation of APL treatment; often combined with As_2_O_3_
Arsenic trioxide (As_2_O_3_)	Degradation of PML-RARA	17.4–29.3	12 (0–46)	Used with ATRA
Ivosidenib	Inhibition of IDH1	11–25	20–29 (1–78)	Fatal post-marketing cases reported
Enasidenib	Inhibition of IDH2	6–19	19–30 (1–150)	Late-onset cases possible
Olutasidenib	Inhibition of IDH1	14–16	18 (1–561)	Late-onset cases possible; rare fatalities
Midostaurin	Inhibition of FLT3	0	NR	AFND observed; no DS cases reported
Gilteritinib	Inhibition of FLT3	1–3	NR (2–75)	DS + AFND in some cases
Quizartinib	Inhibition of FLT3	5	NR	
Revumenib	Inhibition of menin	16–26	18 (5–41)	Grade 2 DS in 16% of patients during early trials
Bexarotene	Retinoid X receptor agonist	5.9	NR	Rarely used in AML
Azacitidine	Hypomethylating agent	8 (alone), 15 (combined with IDH inhibitor)	NR	Lower risk when given alone; higher risk when given in combination with IDH inhibitor

This table is modified and adapted from references [2] and [26]. * The number of days is from the initiation of the drug to the onset of signs and symptoms of DS. When there were multiple reports, the ranges of the median values are stated, and the ranges are combined. AFND, acute febrile neutrophilic dermatosis; AML, acute myeloid leukemia; APL, acute promyelocytic leukemia; ATRA, all-trans retinoic acid; DS, differentiation syndrome; FLT3, FMS-like tyrosine kinase 3; IDH, isocitrate dehydrogenase; NR, not reported; PML-RARA, promyelocytic leukemia-retinoic acid receptor alpha.

**Table 2:** Diagnostic Criteria and Signs or Symptoms of Differentiation Syndrome.

**Classical Diagnostic Criteria for DS (modified from reference [39])**	
Fever	
Dyspnea	
Hypotension	
Rapid weight gain of >5 kg	
Pulmonary infiltrates	
Pleural or pericardial effusions	
Acute kidney injury	
Number of Criteria Present	DS Diagnosis
0	No DS
1	Not sufficient to make a diagnosis
2 to 3	Moderate DS
4 to 7	Severe DS
**Non-classical Signs or Symptoms of DS (modified from references [2] and [26])**	
Leukocytosis	
Dyspnea	
Hypoxia or respiratory distress	
Pericarditis	
Rash	
Lymphadenopathy	
Bone pain or arthralgia	
Disseminated intravascular coagulation	
Edema	
Increased bilirubin levels	
Pancreatitis	
Ocular manifestations (subretinal fluid, macular edema, or exudative hemorrhage retinopathy)	

DS, differentiation syndrome.

**Table 3:** Workup and Management of DS in the Emergency Department.

**Initial Workup for DS**
Medical history/signs and symptoms Patients at risk: those with APL who received induction chemotherapy, including ATRA +/− As_2_O_3_, as well as those with non-M3 AML on differentiating agents Presenting complaints: fever, dyspnea, weight gain, or edema Vital signs, oxygen saturation, and weight Physical examination Full examination with emphasis on the cardiopulmonary system Laboratory tests and imaging studies CBC-differential; complete metabolic panel (including renal function tests and hepatic function tests); coagulation tests; uric acid (especially if renal insufficiency or leukocytosis are present); LDH; magnesium; cardiac panel; BNP/NT-proBNP; infection workup (if febrile), including blood, urine, and sputum cultures and urinalysis Electrocardiogram Imaging studies: chest x-ray, chest CT scan. Consider point-of-care ultrasound if available
**Management of DS**
Ventilatory support/O_2_ supplementation Blood pressure maintenance measures Fluid restriction (renal failure) Steroids: The primary treatment of DS is high-dose glucocorticoid treatment (dexamethasone 10 mg IV every 12 h). This should be started immediately at the time of diagnosis or the earliest clinical suspicion of incipient DS in patients with APL. Antibiotics: Because DS can mimic systemic infections or sepsis, and because it may be impossible to exclude these diagnoses in the ED, it is reasonable to initiate antibiotic therapy, which may be discontinued within 48 h if culture results and imaging studies do not show any evidence of infection. Hold the differentiating agents, which are to be re-started after DS has resolved. Management of leukocytosis. Depending on the level of leukocytosis, cytoreduction with chemotherapy (e.g., hydroxyurea) may be indicated. Avoid leukapheresis or invasive procedures in patients with APL due to the risk of precipitating serious or fatal hemorrhage.

AML, acute myeloid leukemia; APL, acute promyelocytic leukemia; ATRA, all-trans retinoic acid; BNP, brain natriuretic peptide; CBC, complete blood count; CT, computed tomography; DS, differentiation syndrome; ED, emergency department; IV, intravenous; LDH, lactate dehydrogenase; NT-proBNP, *N*-terminal pro-B-type natriuretic peptide.

## Data Availability

No new data were created or analyzed in this study. Data sharing is not applicable to this article.

## Abbreviations

The following abbreviations are used in this manuscript:

AFND	Acute febrile neutrophilic dermatosis
AML	Acute myeloid leukemia
APL	Acute promyelocytic leukemia
ATRA	All-trans retinoic acid
BNP	Brain natriuretic peptide
CBC	Complete blood count
CCL	C-C motif chemokine ligand
CTCAE v5	Common Terminology Criteria for Adverse Events version 5.0
CT	Computed tomography
DS	Differentiation syndrome
ED	Emergency department
FLT3	FMS-like tyrosine kinase 3
ICAM-2	Intercellular adhesion molecule 2
ICC	International consensus classification
IDH	Isocitrate dehydrogenase
IL	Interleukin
IV	Intravenous
KMT2A	Lysine methyltransferase 2a
LFA-1	Leukocyte function-associated antigen 1
MLCK	Myosin light chain kinase
NF-κB	Nuclear factor-kappa B
NR	Not reported
NT-proBNP	*N*-terminal pro-B-type natriuretic peptide
NUP98	Nucleoporin 98
PML-RARA	Promyelocytic leukemi-retinoic acid receptor alpha
RhoA	Ras homolog family member A
RXR	Retinoid X receptors
SIRS	Systemic inflammatory response syndrome
TET	Ten-eleven translocation
TG2	Transglutaminase 2
TNFalpha	Tumor necrosis factor alpha
VEGF	Vascular endothelial growth factor
WBC	White blood cell
WHO	World Health Organization
